# Case report: Pacemaker lead perforation of a papillary muscle inducing severe tricuspid regurgitation

**DOI:** 10.1186/s13019-015-0244-7

**Published:** 2015-03-25

**Authors:** Martin Andreas, Franz Gremmel, Andreas Habertheuer, Claus Rath, Claudia Oeser, Cesar Khazen, Alfred Kocher

**Affiliations:** Department of Cardiac Surgery, Medical University of Vienna, Waehringer Guertel 18-20, Level 20A, 1090 Vienna, Austria

**Keywords:** Pacemaker complication, Lead perforation, Lead extraction, Papillary muscle

## Abstract

**Introduction:**

We report a rare but severe pacemaker complication of a pacemaker lead perforating the papillary muscle. This induced severe tricuspid regurgitation and right heart failure. Patients suffering from right heart failure have an increased operative risk of open-heart surgery and therefore represent a clinical challenge due to the lack of clear guidelines.

**Case presentation:**

A 70-year-old male patient presented with severe tricuspid regurgitation and a history of decompensated right heart failure. One pacemaker lead was described as ‘whipping’. Four years earlier he had received a VVIR pacemaker with a passive lead. This lead failed after three years and a new ventricular lead had been placed. We performed on-pump beating heart surgery after a multidisciplinary decision process. One lead was perforating the posterior papillary muscle, severely impairing valve movement. The tricuspid valve was replaced with a stented bioprosthesis. Epicardial pacemaker wires were placed on the right and left ventricle to enable cardiac resynchronization therapy in the case of postoperative heart failure. However, the patient recovered quickly without left ventricular pacing and could be discharged home 12 days after surgery.

**Conclusion:**

This particular case emphasizes the importance of meticulous surgical technique during pacemaker lead implantation and a tight postoperative follow-up including echocardiography in complicated cases. The management of patients with an indication for lead removal having developed secondary severe tricuspid valve dysfunction inducing ventricular impairment represents a clinical challenge and should be approached by a multidisciplinary team.

**Electronic supplementary material:**

The online version of this article (doi:10.1186/s13019-015-0244-7) contains supplementary material, which is available to authorized users.

## Background

The number of pacemaker implantations is still on the rise with a broad variety of techniques described and devices available. Complications are not rare and meticulous attention has to be paid to avoid potential risks and to identify and treat all kinds of adverse events [1]. Special techniques were recently developed to remove malfunctioning or infected leads by transvenous lead extraction devices [2]. However, these techniques have their limitations and may also lead to severe adverse events requiring urgent surgical intervention. Therefore, pacemaker specialists performing lead extraction have to be familiar with transvenous and open-heart surgical techniques.

This case reports a pacemaker lead perforating a papillary muscle. Lead-induced valve dysfunction may induce ventricular failure years after lead implantation. Once ventricular dysfunction has developed, the risk of surgical procedures increase both with regard to perioperative morbidity and mortality and the therapeutic options are limited. Clear guidelines are lacking in these patients, which is especially true for patients with right heart failure. Therefore, we report this case of an effective surgical approach in a patient with right heart failure to provide guidance in this complex clinical setting.

## Case presentation

A 70-year-old male patient was admitted to the department of cardiac surgery for surgical repair of severe tricuspid regurgitation. He had a history of decompensated right heart failure with recurrent hospitalizations. Four years earlier he had received a VVIR pacemaker with a passive lead (Refino 58 ER, Oscor; Palm Harbor, FL, USA) due to atrial fibrillation with atrioventricular conduction block. Three years later, another passively fixed ventricular lead had been placed due to a failure of the original pacemaker lead resulting in increasing impedance.

**Figure 1:**
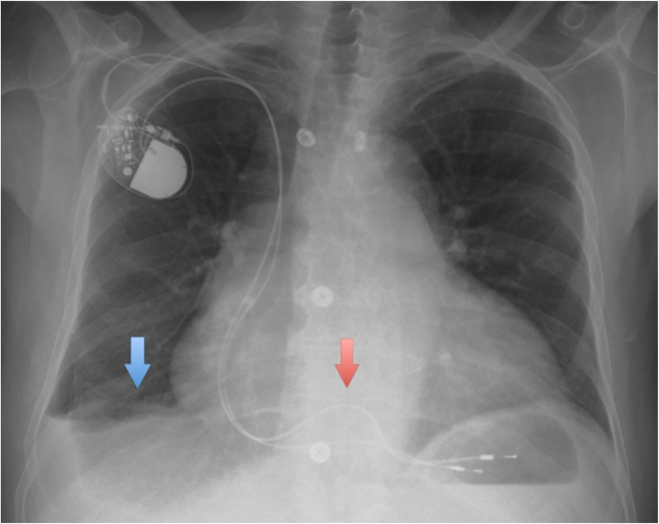
**Preoperative chest X-ray.** Blue arrow: pleural effusion; Red arrow: different course of the two leads through the tricuspid valve.

The chest X-ray at admission revealed a significantly enlarged heart and pleural effusions due to right heart failure (Figure 1). The pacemaker leads did not show a parallel course through the tricuspid valve. Transthoracic echocardiography confirmed severe tricuspid regurgitation with a moderate impairment of right ventricular function (Additional file 1: Video S1). The left ventricle and the other heart valves did not show any pathologic findings. However, one pacemaker lead was described as ‘whipping’ in the transthoracic echocardiography (Additional file 1: Video S1).

Clinical decision-making was guided by a multidisciplinary approach. Options considered were a conservative approach due to the increased risk of postoperative right heart failure, lead removal by percutaneous lead extraction or open surgical lead removal including valve repair or replacement. Eventually the decision was taken for an open surgical approach due to the patient’s good general health condition and the severely dilated tricuspid annulus.

**Figure 2:**
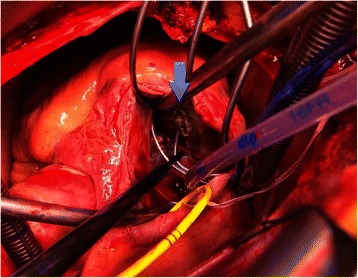
**Intraoperative view of the perforating lead.** Blue arrow: pacemaker lead perforating the papillary muscle.

**Figure 3:**
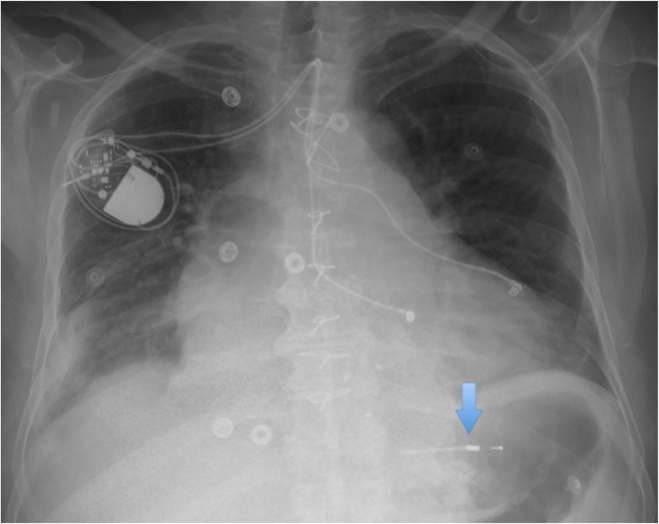
**Postoperative chest X-ray.** Blue arrow: residual lead fragment.

On-pump beating heart surgery was performed. One lead was perforating the posterior papillary muscle, severely impairing valve movement (Additional file 1: Video S1, Figure 2). Although the lead was not perforating the leaflet itself, it prevented the free margin of the posterior leaflet to close in systole due to its strong adhesion to the papillary muscle and the ventricular wall. The leaflet itself was not altered. The tip could not be completely removed, and can still be seen on the postoperative chest X-ray (Figure 3). The second lead could be removed without problems. The valve was reconstructed with a Contour 3D tricuspid annuloplasty ring (Medtronic Inc., Minneapolis, MN, USA). However, the reconstructed valve showed again moderate regurgitation after weaning from the heart-lung machine and was replaced with a stented bioprosthesis (Mosaic Ultra Mitral, Medtronic Inc., Minneapolis, MN, USA). Epicardial leads were placed on the right and left ventricle. The lead to the right ventricle was connected to the pulse generator. The lead to the left ventricle was routed to the pacemaker pocket to allow cardiac resynchronization therapy in the case of postoperative heart failure. However, the patient could be weaned from cardiopulmonary bypass without need for mechanical support or resynchronization. Postoperative valve function was good and inotropic support could be stopped after four days. The left ventricular lead has not been connected due to sufficient left ventricular function and the absence of heart-failure symptoms. The patient was discharged home 12 days after surgery in good condition and was stable thereafter.

## Discussion and conclusion

This particular case highlights the importance of meticulous surgical technique during pacemaker lead implantation. Perforations of the leaflet and the papillary muscle may be avoided by the prolapsing technique. A loop of the lead is formed by positioning the tip in an area of the right atrium. Thereafter, the loop rather than the tip of the lead is advanced into the right ventricle [3]. By that, a puncture of the valve leaflet or the papillary muscle is avoided and the coronary sinus cannot be entered by mistake. Furthermore, a tight postoperative follow-up including echocardiography should be applied in complicated cases. Impairment of tricuspid function after right ventricular lead implantation is not uncommon [3]. A recent analysis associated right ventricular lead implantation with increased tricuspid regurgitation, higher pulmonary artery pressure and a dilation of the right ventricle [4]. Further, a significant lead-induced tricuspid regurgitation was associated with a poor prognosis. A potential alternative in patients developing tricuspid regurgitation early after pacemaker implantation would be to replace the right ventricular lead by a left ventricular lead placed through the coronary sinus and to remove the right ventricular lead thereafter.

The lead’s whipping movement should be a warning sign of lead entrapment in the valvular apparatus. Furthermore, the management of patients with an indication for lead extraction having developed secondary severe valve dysfunction inducing ventricular impairment and right-sided heart failure represents a clinical challenge requiring a multidisciplinary approach. Although an increase in tricuspid regurgitation is not common after lead extraction, the use of powered sheath-assisted extraction, which would have been necessary in this case, was associated with worsening of valve function [5]. We strongly believe that lead extraction alone would not have provided significant benefit in this late disease state. Improved valve function after extraction was unlikely due to severe annular dilation.

Therefore, we highlight the importance of surgical intervention including valve reconstruction or replacement in severely symptomatic high-grade tricuspid regurgitation prior to the development of irreversible right heart failure according to current guidelines in contrast to lead extraction techniques or heart failure therapy alone [6].

## Consent

Written informed consent was obtained from the patient for publication of this case report and any accompanying images. A copy of the written consent is available for review by the Editor-in-Chief of this journal.

## Additional file

Additional file 1: Video S1. Preoperative echocardiography and intraoperative view. The echocardiographic loops show the ‘whipping’ movement of the entrapped lead, the high-grade tricuspid regurgitation and the intraoperative view.
